# Editorial: New insights in diagnosis and therapy of hormone-dependent cancer

**DOI:** 10.3389/fendo.2022.1115341

**Published:** 2023-01-10

**Authors:** Monica Rienzo, Cristina Pagano, Felice Crocetto, Erika Di Zazzo

**Affiliations:** ^1^ Department of Environmental, Biological, and Pharmaceutical Sciences and Technologies, University of Campania “Luigi Vanvitelli”, Caserta, Italy; ^2^ Department of Molecular Medicine and Medical Biotechnology, University of Naples “Federico II”, Naples, Italy; ^3^ Department of Neurosciences, Reproductive Sciences and Odontostomatology, University of Naples Federico II, Naples, Italy; ^4^ Department of Medicine and Health Sciences “V. Tiberio”, University of Molise, UOC Laboratorio Analisi P.O. “A. Cardarelli”, Campobasso, Italy

**Keywords:** hormone-dependent cancers, prostate cancer, breast cancer, endometrial cancer, prognosis, CtDNA, biomarkers, metastasis

Hormone-dependent cancers, including breast, ovarian, uterine, and prostate cancers contribute to most cancer incidence and death worldwide. In the last few decades, considerable efforts have been made to discover new biomarkers and therapeutic options to fight hormone-dependent cancers. Although advances in early diagnosis and detection have been obtained, numerous late diagnoses occur. Moreover, the therapies currently available often fail and patients develop drug resistance and relapse is frequently observed (1). Prostate cancer (PC) is a common malignancy in Western society, representing the most diagnosed cancers and a cancer-related death leading cause. Compelling evidence has shown that steroids and their receptors are associated with PC pathogenesis, controlling its initiation and progression (2, 3; Rossi et al.). In order to discover novel predictors of PC invasion, Wang et al., by performing a multivariate regression analysis demonstrated that red cell distribution width (RDW) and neoadjuvant hormonal therapy were negatively correlated with lymphovascular invasion (LVI), while PSA levels, international society of urological pathology (ISUP) grade, and pathological stage T (pT) were positively correlated with LVI in patients who underwent radical prostatectomy (RP). Similarly, two nomogram models evaluating the status of axillary lymph nodes-ALNs (N0, 1-2 positive ALNs or >2 positive ALNs) were constructed to accurately predict breast cancer (BC) metastasis in BC patients (cN0) (Gao et al.). This evidence could help in making more accurate therapeutic options and, overall, improve the cancer management. Prognostic biomarkers aim to evaluate the patient’s overall outcome and the cancer recurrence probability after standard treatment. The current Research Topic focuses the attention on novel biomarkers that could improve diagnosis and prognosis. Wang et al., by performing a multivariate Cox regression analysis discovered a novel ferroptosis-related gene prognostic index (FRGPI) based on four genes (E2F1, CDC20, TYMS, and NUP85) that can predict the disease-free survival (DFS) and response to immunotherapy for PC patients after RP. Moreover, Fu et al., developed a novel pyroptosis-related signature based on twelve genes (CASP8, GSDMB, BAK1, BAX, CHMP4B, CHMP4C, CHMP6, TP53, TP63, CASP9, GPX4 and PLCG1) to assess PC prognosis and recurrence free survival (RFS) by integrative analyses of bulk and single-cell RNA sequencing (RNA-seq) profiling followed by RT-qPCR and immunofluorescence validation. In addition, prognostic biomarkers can be useful for the selection of patients eligible for a therapeutic option. In this context, a nomogram was developed to predict the survival for stage IIIC endometrial cancer (EC) patients treated with adjuvant radiotherapy (ART) alone or followed by adjuvant chemotherapy (ACT) (Yang et al.).

Recently, liquid biopsy has emerged as an attractive and promising strategy complementary to invasive tissue biopsy. Several blood biomarkers, including circulating tumor cells (CTCs), extracellular vesicles (EVs), circulating tumor DNA (ctDNA) and RNA (ctRNA), could be analyzed for diagnostic, prognostic and predictive purposes, as well as for therapy response monitoring (4). ctDNA analysis by next generation sequencing (NGS) revealed that the top ctDNA altered genes TP53, PIK3CA helix domain mutation (PIK3CA-HD), FGFR, ESR1 and GATA3 were related to endocrine therapy -resistance in metastatic BC (mBCs) patients. The performed analysis successively guided the therapy. Kaplan–Meier curves, indeed, showed that patients that received druggable ctDNA -guided late-line therapy had significantly longer PFS than those who received physician-chosen therapy (Tang et al.). In the last few years, novel therapeutic approaches have been proposed, spanning form immunotherapy-based ones to cell cycle inhibitors (5, 6). Particularly, several cyclin- dependent kinase (CDK)4/6 inhibitors have emerged as novel therapeutical approaches for BC management. BC is a heterogeneous disease and effective therapies are elusive for the more aggressive subtype, triple negative BC (TNBC). Among them, hormone-receptor (HR)-positive and/human epidermal growth factor receptor 2 (HER2) HER2-negative mBCs become resistant to endocrine therapy after early-line (first- and second-line) endocrine therapy (Giovannelli et al., 7). Recently, accumulating evidence supports that a novel distinct HR positive/HER2-low BC subtype with specific clinicopathological features exists. However, Shao et al., revealed that HER2-low expression does not affect the clinical efficacy of CDK4/6 inhibitors.

In conclusion, all the collected manuscripts in this special issue add novel insights on PC, BC and EC. In addition, this collection reveals that deepen the knowledge of hormone-dependent cancers could help the precision medicine to develop personalized and efficacious therapies to cure the most common hormone-dependent cancers. However, additional attempts are needed to improve early diagnosis, follow-up and predict prognosis.

## Author contributions

EDZ contributed to the conception of the draft. EDZ and MR wrote the first draft. EDZ supervised. All authors contributed to manuscript revision, read, and approved the submitted version.
